# Hospitals and Vaccine Therapy

**Published:** 1914-01-24

**Authors:** 


### HOSPITALS AND VACCINE THERAPY.

A remarkable address on the subject of vaccine treatment is contributed to the *Medical Chronicle* for December by Dr. H. Batty Shaw, physician to University College Hospital, being the annual address delivered before the Norwich Medico-Chirurgical Society in October. The author concedes that vaccines have the highest value as prophylactics for many diseases; and he is also prepared to admit that in some cases remarkable improvements are shown under vaccine treatment of diseases already existing. But he emphasises the uncertainty of this latter kind of vaccine treatment, and especially the jumping to conclusions as to cause and effect without ample control cases.

He points out also that the introduction of the successful serum treatments—as, for instance that of diphtheria—was preceded by ample experimental work on animals; whereas vaccines have been comparatively little tested in this way. "The possibility of spontaneous cures does not enter into the domain of thought of the ordinary vaccinist," says Dr. Shaw, and he goes on to define the quarrel of the thoughtful clinician with the vaccinist. The clinician admits, or ought to admit, merely that he relieves symptoms: not even salicylates for rheumatism, colchicum for gout, nor mercury for syphilis are true causative cures. But the vaccinist claims to treat the causes of disease, and it is this claim that Dr. Shaw scrutinises with a jealous eye. He quotes, as a lesson, a case of exceedingly severe malignant streptococcal endocarditis diagnosed bacteriologically as well as clinically, in which, after other treatment failed, a polyvalent serum was ordered. Within three days the temperature began to fall, and after three more it was normal. The physician was about to publish the case when, to his dismay, he discovered that, owing to a misunderstanding, the serum had never been given at all!

The recent work of Wolff-Eisner and Friedberger explains to Dr. Batty Shaw's satisfaction why vaccines are so variable in their effects, and why those effects are sometimes disastrous instead of beneficial. The discussion of these questions is highly technical, and need not detain us now. From an institutional aspect the matter is chiefly of importance from the fact that for the last few years every hospital of size and standing has felt bound, by the pressure of medical and lay opinion, to appoint a vaccinist medical specialist, and to spend a lot of money on laboratories and materials for his work. Mr. Granville Barker's revival of "The Doctor's Dilemma" reminds us of the clever mockery of vaccines and the craze for them which forms one of the leading motives of that play. If Dr. Shaw is right, his namesake, the playwright, was not far off the track in satirising the Bloomfield Bonningtons and Colenso Ridgeons of the day. G. B. S. would, we suppose, protest that diphtheria anti-toxin, anti-typhoid preventive vaccination, and other processes founded on experimental research are equally nonsensical; but he will find no support whatever for his pet propaganda in Dr. Batty Shaw's article. Rather the latter favours the continuation of research so that the faults which he sees in some of the current methods may be eliminated. Vaccines, he declares, should be abandoned in human therapeutics until it has been demonstrated upon animals that by vaccines all invading organisms can be killed off, and yet at the same time the host suffer no harm by anaphylatoxins. If ever there was a problem worthy of the steel of a practised bacteriologist, here it is. Vaccinists have been so busy applying their vaccines to human patients that they have failed to test their theories first of all on animals. Meanwhile clinicians who are dissatisfied with the alleged benefits of vaccine treatment may with reason withhold vaccine therapy. Evidence is so far not forthcoming that vaccination after infection has occurred leads to the destruction of the infecting micro-organisms.

There is, of course, another side to this case: that of those who have faith in vaccines. Nevertheless such scepticism as Dr. Shaw's is healthy, and we should like to see Sir Almroth Wright answer his criticisms.

